# Subjective working memory predicts objective memory in cognitively normal aging: a HUNT study

**DOI:** 10.1186/s40359-020-00447-9

**Published:** 2020-07-29

**Authors:** Ole Bosnes, Ove Almkvist, Ingunn Bosnes, Eystein Stordal

**Affiliations:** 1 Namsos Hospital, Nord-Trøndelag Hospital Trust, Namsos, Norway; 2 Division of Clinical Geriatrics, Department of Neurobiology Care Sciences and Society, Karolinska Institutet, Stockholm, Sweden; 3 Department of Psychology, Norwegian University of Science and Technology (NTNU), Trondheim, Norway; 4 Department of Mental Health, Norwegian University of Science and Technology (NTNU), Trondheim, Norway

**Keywords:** Subjective memory tests, Objective memory tests

## Abstract

**Background:**

Recent studies have shown that subjective memory is multi-, rather than uni-dimensional, in line with the results of objective memory tests. The purpose of this study was to investigate whether there is an association between aspects of memory measured by the subjective Meta-Memory Questionnaire (MMQ) and aspects of memory measured by the objective Wechsler Memory Scale-III (WMS-III) and Wechsler Adult Intelligence Scale-III (WAIS-III) tests in cognitively normal older adults.

**Method:**

The study subjects (*n* = 106) were cognitively normal, were aged 57–89 years and had participated in the third wave of the North-Trøndelag Health survey (HUNT3). All subjects had completed the MMQ, the WMS-III and the WAIS-III. Previous results from the MMQ (measured as the total MMQ score; the Component I score, related to long-term explicit declarative memory; and the Component II score, related to working/short-term memory) were compared with objective results from WMS-III (Logical Memory) and WAIS-III (Vocabulary and Letter-Number Sequencing) subtests. We conducted linear regression analyses with each objective memory test result as the dependent variable, and subjective memory measures and demographics as independent variables, as well as analyses of MMQ items vs. objective memory.

**Results:**

Subjective working memory impairment (Component II) was significant related to poor performance in objective episodic memory, according to correlation and regression analyses with demographic covariates. In contrast, ratings of impaired subjective declarative memory (Component I) were not related to poor objective memory performance.

**Conclusions:**

Specific aspects of subjective memory related differentially to performance in specific objective memory tests. Clinicians and researchers might consider targeting working memory aspects of subjective memory tests, when seeking an estimate of objective memory performance.

## Background

Self-reported memory complaints are common in older adults [1] and the prevalence increases with age, according to population- [2–4] and community-based studies [5, 6]. Previous research has demonstrated that the relationship between subjective memory test results and objective memory performance is complex and unclear [7]. Individuals complaining of memory problems may have no objective signs of memory impairment [8, 9]. On the other hand, memory complaints may be a preclinical sign of dementia in individuals with verified cognitive decline and memory impairment, exemplified by mild cognitive impairment (MCI) [10]. Many of those with clinically diagnosed dementia and objective memory impairment do not complain of memory loss, presumably because they lack insight into their serious impairment [11, 12]. Previous research has suggested that the lack of a consistent relationship between subjective and objective memory performance may be related to non-memory factors such as depression [1], neuroticism [13, 14], poor somatic health [15] and health status (healthy or not healthy individuals). There are other important reasons for the seeming lack of relationship between subjective and objective memory performance, including use of different sample populations [16], limitations of the cognitive tests used [17], and the measurement of different aspects of cognitive ability by the subjective and objective memory tests [18]. At a personal level the authors have often met older adults claiming about memory problems, but who perform well on objective memory tests, while perhaps showing some problems with attention. To summarize, although a meta-study [11] concluded that it is a small positive correlation (*r* = 0.15) between subjective and objective memory performance, the association between subjective and objective memory performance are only partly understood.

Furthermore, research in the field is seriously hampered by the lack of a commonly accepted definition of memory from a subjective point of view and the poor quality of subjective memory testing methods [19, 20]. An extensive review of subjective memory questionnaires, which listed a total of 34 self-reported measures used in 19 studies in different countries, stated that the majority of instruments did not document satisfactory statistically assessable characteristics (e. g. reliability, validity and factor structure), which questions the use of the instruments in research and clinical practice [17]. In addition, the questionnaires differed widely in type, content and number of items, which makes it hard to compare the results. This may indicate that tests of subjective memory have more than one dimension and that single sum scores from such tests therefor may be oversimplifying and partly be the cause of divergent findings.

The Meta-Memory Questionnaire (MMQ) was chosen as a test of subjective memory in the present study, because it has been used previously in the Nordic countries [21–25], the internal structure has been investigated and found to be multi-dimensional [20], because there is no international standard of subjective memory test to rely on [17], and because this nine item questionnaire was used in the third wave of the North-Trøndelag Health survey (HUNT3) [26]. Interestingly, six among the nine items in the MMQ are included in the list of the 10 most common items in other questionnaires that assess subjective memory as reported by Rabin and associates [17]. Three items in MMQ (recent events, conversation and intention) related to working memory (component II) and three items in MMQ (general memory, memory change and names) related to declarative memory (component I) were common in other subjective memory instrument. This commonality makes it possible to generalize the present findings to subjective memory questionnaires in general.

The HUNT study (an acronym for the Norwegian name: **H**else**u**ndersøkelsen i**N**ord-**T**røndelag), which by now has been completed four times from 1984 to 2018, is a large population based study, covering a wide range of medical, social and health related data of all inhabitants in the county of Nord-Trøndelag in Norway (about 125,000). The surveys have a high participation rate and data drawn from samples in the surveys may therefore be generalizable to the population in Norway. Detailed information about the surveys is found at the HUNT homepage [26]. A previous study of subjective memory performance based on the total MMQ score in HUNT3 found that approximately 45% of the participants reported minor memory problems and 1.5% reported major memory problems, and that men reported more memory impairment than women [22]. That study assessed subjective memory performance using a single measure (total score).

In the present study subjective memory performance was assessed also using the two components of the MMQ detected in a previous study [20]: one named Component I, associated with long term declarative memory (5 items about memory problems, changed memories, remembering names, dates and events from years ago), and another factor named Component II, associated with working/short-term memory and linked to attention (4 items about events from minutes or days ago, planned activities, and keeping track in a conversation). The reliability, in terms of PCA structure, showed selectively high associations (r > 0.80) between 5 items and Component I, and 4 items and Component II [20].

Whether the MMQ total score or either of its components is valid indicators of objective memory performance has not previously been studied, although this knowledge would enhance the utility of the instrument significantly and be of great interest, both to clinicians and researchers using the MMQ and similar questionnaires. This explorative study aims to fill this gap.

We investigated the relationships between subjective memory performance, expressed as the total score, the component scores and scores on single items of the MMQ, and performance on objective memory tests in a sample of cognitively normal older adults, while also considering the effects of demographics (age, gender and level of education) as covariates, because it is well-known they are important factors for cognition in general and for memory in particular. As an exploratory study no precise hypothesis were stated, but we anticipated that the three scores derived from the MMQ (the total score and the scores of Components I and II) were differentially related to objective memory performance. If so, this would have important implications on how to make empirically based interpretations of scores on the MMQ, which is the main reason for this study.

## Methods

### Participants

Participants in the present study (*n* = 106) were extracted from a sample of community-dwelling older adults who had participated in a study of memory and intelligence conducted in 2009 and 2010 [27], and had complete MMQ data in HUNT3, conducted in 2006–2008 [26]. Inclusion criteria in the study of memory and intelligence were (i) age 55–89 years, (ii) self-reported good health and daily functioning according to two questionnaires in HUNT3 (Q1 and Q2, [26]), (iii) living near the city of Namsos in middle Norway (the examination site), and (iv) confirmed unchanged cognition since HUNT3 by a structured clinical interview before testing. Two individuals were excluded because their health was changed. Health was checked by using a similar procedure as used in the standardization study of the Wechsler Adult Intelligence Scale-III and the Wechsler Memory Scale-III (WAIS-III/WMS-III), for details see [27]. Education data were divided into three levels: elementary school, high school, and college/university. The demographic characteristics of the study sample and eligible participants are presented in Table 1, which shows that the mean age of the study sample was significantly higher than that of the comparable group in HUNT3 (t = 8.36 df = 105, *p* <  0.001), while the education levels and gender distributions were comparable (p’s > 0.1). The comparison of the study sample and the population will help facilitate generalization of results.

**Table 1:** Demographic characteristics of the study sample and of eligible participants from HUNT3

	Study sample	HUNT3
N	106	17,247
Age (Mean ± SD), y	73.4 ± 8.4	66.6 ± 8.7
Range, y	57–89	55–101
Gender, frequency of females (%)	53 (50)	8968 (52)
Education		
Elementary school, n (%)	47 (44)	7029 (41)
High school, n (%)	34 (32)	6086 (40)
College/university, n (%)	25 (24)	3332 (19)

### Subjective and objective memory assessment

Memory was assessed subjectively in HUNT3 [26] using the MMQ, which contains nine items for assessing memory problems. Each item is scored out of 3 (1 = no problems, 2 = sometimes problems, and 3 = often problems). The usual approach is to sum the individual item scores to obtain the total score (range 9–27), where a score of 9 means no problems and a score of 27 means severe impairment. An earlier principal component analysis showed that the nine items of the MMQ can be described by two largely independent components covering explicit, long-term declarative memory and working/short-term memory related to attention (as discussed in the introduction; see [20] for details). We included the scores from the two MMQ components in this study, in addition to the commonly reported total score, as possible predictors of objective memory performance.

Data on objective memory performance and intelligence were collected in a study of the psychometric characteristics of the Norwegian versions of the WMS-III and WAIS-III [27, 28]. We used the scores from three subtests of memory: Logical Memory I (LM) in the WMS-III to study new auditory verbal learning and recall (episodic memory), Vocabulary (Voc) in the WAIS-III to study recall of overlearned verbal material (semantic memory) and Letter-Number Sequencing (LNS) in the WAIS-III to study auditory working memory. These subtests were chosen because they represent different aspects of objective memory performance according to theories about memory [29]. Because research on the factor structure of the WMS-III has demonstrated that the immediate (LM I) and delayed recall (LM II) tests from the WMS-III are associated to the same factor [28, 30], LM II was not included in the present study.

### Statistical analyses

Descriptive statistics and correlation analyses (Pearson and Kendall’s tau) was used to characterize the sample and associations. To analyze the relationship between each objective memory test and subjective memory variables (Component I, Component II and demographic variables), multiple regression analyses was used by entering predictors simultaneously. This procedure was repeated with MMQ total score and demographics as predictors. There was no problem with multicollinearity in the regression analyses as tolerance values were close to 1 for variables in the analyses. Health status was not included in the analyses, because the inclusion criteria ensured that the sample included older adults who had good daily functioning and were free of major diseases and disabilities. To check for the influence of the varying time interval between HUNT3 and cognitive testing, age-adjusted results for objective memory were calculated and used in regression analyses. The outcome of these analyses did not differ from those presented in Table 5 (based on un-adjusted test scores) and the accompanied text: significant predictors were the same as well as multiple r’s and beta weights (second decimal differed sometimes).

## Results

The analyses of subjective memory performance focused on the total MMQ score, the scores of the MMQ components, and the single item MMQ scores.

### Subjective memory performance

The mean MMQ total and Component II scores were lower than in the corresponding HUNT3 population, but not significantly so, see Table 2.

**Table 2:** Total raw score MMQ and z scores of Components I and II (means ± SD) of the study sample and the HUNT3 population (across six age groups)

	Age group					
MMQ score	55–64 *n* = 17	65–69 *n* = 19	70–74 *n* = 19	75–79 *n* = 21	80–84 *n* = 18	85–89 *n* = 12
Normally aging adults						
Total	12.7 ± 1.9	14.2 ± 2.5	14.7 ± 1.9	14.2 ± 2.7	15.1 ± 3.6	13.3 ± 2.6
Component I	−0.04 ± 0.91	− 0.10 ± 0.63	0.48 ± 0.57	0.11 ± 0.87	0.26 ± 1.24	−0.1 ± 0.85
Component II	− 0.67 ± 0.61	−0.08 ± 1.04	−0.24 ± 0.71	−0.08 ± 1.12	0.17 ± 0.90	−0.22 ± 0.83
HUNT population						
Total	14.3 ± 3.0	14.8 ± 2.9	14.7 ± 3.0	15.2 ± 3.1	15.3 ± 3.3	15.8 ± 3.9
Component I	0.12 ± 0.96	0.27 ± 0.92	0.20 ± 0.91	0.30 ± 0.94	0.26 ± 0.95	0.26 ± 1.07
Component II	− 0.04 ± 1.00	0.01 ± 1.04	0.07 ± 1.06	0.20 ± 1.11	0.28 ± 1.16	− 0.52 ± 1.28

The MMQ scores for the six studied age groups are presented in Table 2, along with the MMQ scores from the HUNT3 population. The cognitively normal older adult group, the oldest members in particular, reported fewer memory problems than the HUNT3 population.

The correlation coefficients between age and MMQ measures, gender and MMQ measures, and level of education and MMQ measures were not significant for any association (all p’s > 0.1).

### Objective memory performance

The mean raw scores for the WMS-III LM subtest (episodic memory), and the WAIS-III Voc (semantic memory) and LNS (working memory) subtests across the six age groups are presented in Table 3. The LM scores in the study sample were higher than the norms published in the WAIS-III and WMS-III manuals, but not significantly higher [30], whereas the Voc and LNS scores were similar to the norms.

**Table 3:** Raw scores (means ±SD) on LM, Voc and LNS across age groups in cognitively normal participants

	Age group					
Test	55–64 *n* = 17	65–69 *n* = 19	70–74 *n* = 19	75–79 *n* = 21	80–84 *n* = 18	85–89 *n* = 12
LM I	42.6 ± 8.4	39.2 ± 10.5	35.3 ± 12.8	31.1 ± 11.5	32.4 ± 9.7	28.5 ± 9.5
Voc	45.4 ± 9.3	40.5 ± 8.4	42.1 ± 11.9	35.0 ± 10.0	33.7 ± 10.0	26.8 ± 7.9
LNS	9.1 ± 2.2	7.7 ± 2.2	8.1 ± 2.7	7.1 ± 1.6	7.1 ± 1.5	5.5 ± 1.9

The mean scaled scores for the study sample were normally distributed (Means ± SD: LM 10.7 ± 3.3, Voc 9.7 ± 2.5 and LNS 9.5 ± 2.4) and close to the US norms in the WAIS-III and WMS-III manuals [28].

The Pearson correlation coefficients for the association between age and the three objective memory tests were clearly significant (LM *r* = − 0.39, Voc *r* = −.049 and LNS *r* = −.041; all *p* <  0.001). The associations between level of education and the three objective memory tests were also clearly significant (LM *r* = 0.37, Voc *r* = 0.75 and LNS *r* = 0.50; all *p* < 0.001). There was no significant association between gender and the three objective memory tests.

### Association between subjective and objective memory performance

The correlations between the MMQ total and component scores and the objective memory scores are presented in Table 4. The correlations between subjective and objective memory scores were analyzed using the Pearson correlation coefficient and the t-test with 104 degrees of freedom. The total MMQ score was significantly associated with the LM score. Component I was not significantly associated with any of the three objective memory measures, whereas Component II was significantly negatively correlated with all three measures. Because there was a varying time interval between the MMQ assessments and the objective memory tests, the correlation analyses were repeated to control for the time interval. The same pattern of results remained.

**Table 4:** Correlation coefficients for MMQ total and component scores, versus LM, Voc and LNS raw scores

Test	MMQ total	Component I	Component II
LM	−0.27**	−0.05	− 0.33***
Voc	−0.06	0.12	−0.21*
LNS	−0.01	0.18	−0.22**

* = *p*<0.05; ** = *p* < − 0.01; *** = *p* < 0.001. Significant results are bolded

### Predictors of objective memory performance

We conducted a number of linear regression analyses for each objective test, see Table 5.

**Table 5:** Multiple regression analyses on LM, Voc and LNS with components, age, gender and education as predictors

Independent variables	*B*	*SE B*	β	p
Logical Memory				
Age	−.32	1.11	−.22	0.022
Gender	−1.92	2.08	−.09	0.348
Education	4.06	1.41	0.28	0.005
Component I	−0.67	1.11	−.0.05	0.560
Component II	−3.25	1.11	−0.26	0.004
Vocabulary				
Age	−0.23	0.09	−0.16	0.018
Gender	1.30	1.43	0.06	0.385
Education	9.17	0.98	0.66	< 0.001
Component I	1.69	0.77	0.13	0.031
Component II	−1.83	0.77	−0.15	0.019
Letter-Number Sequencing				
Age	−0.06	0.26	0.20	0.028
Gender	0.03	0.39	0.01	0.957
Education	1.15	0.27	0.41	< 0.001
Component I	0.48	0.21	0.18	0.026
Component II	−0.36	0.21	−0.15	0.085

Firstly, LM performance was used as the dependent variable in relation to the following independent variables: age, gender, level of education, and the two MMQ components. This model was significant (F = 7.83 (5, 100), *p* < 0.001, *multiple r* = 0.53, *r*^2^ = 0.28) and explained 25% (*adjusted r*^2^) of the variance in LM with three significant predictors: age, education and Component II (negative beta). Component I and gender were not significant predictors.

Next, the analysis was repeated with Voc as the dependent variable, and the same independent variables as above. The model was significant (F = 35.57 (5, 100), *p* < 0.001, *multiple r* = 0.80, *r*^2^ = 0.64), with age, education, Component I (positive beta) and II (negative beta) as significant predictors. This model accounted for 62% (*adjusted r*^2^) of the total variance in Voc.

A corresponding analysis was performed with LNS as the dependent variable and the same predictors as above. The model was significant (F = 10.72 (5, 99), < 0.001, *multiple r* = 0.59, *r*^2^ = 0.35) with three significant predictors: age, education and Component I (positive beta), accounting for 32% (*adjusted r*^2^) of the variance in LNS.

Then the analyses were repeated with each objective memory test score as the dependent variable and the total MMQ score, age, gender and level of education as independent variables. The total MMQ score was a significant predictor of LM (F = 8.73 (4, 101), *p* < 0.001; *multiple r* = 0.51, *r*^2^ = 0.26) together with age and education as significant predictors (β = − 0.24, *p* = 0.013; β = − 0.20, *p* = 0.025; age and education, respectively) accounting for 23% of the variance (*adjusted r*^2^). In contrast, the total MMQ score was not a significant predictor for the two other objective memory tests in the regression analyses (*p* = 0.937 and *p* = 0.553; Voc and LNS, respectively).

### Sensitive items in subjective memory measurement

Table 6 shows the Pearson partial correlations between the single MMQ items and the age-adjusted objective memory test scores at HUNT3, controlling for age, gender and education level. Three MMQ items (3, 7 and 9) linked to Component II were significantly associated with LM performance and item 9 (“Do you have problems keeping track of a conversation?”) was linked to Voc performance. In addition, item 3 (“Do you have problems remembering what happened a few minutes go?”) was significantly associated with LNS performance. In contrast, no item was significantly linked with Component I. The positive correlation between item 8 (“Do you have problems remembering events years ago?”) and LNS was unreasonable because of the direction (the more impairment the better the performance).

**Table 6:** Pearson correlation coefficients for MMQ items tapping Components I and II versus LM, Voc and LNS

	Component Item	LM	Voc	LNS
I	1 Memory problems	−0.18	−0.02	0.00
I	2 Changed memory	0.03	0.16	0.10
I	4 Remembering names	−0.07	0.15	0.12
I	5 Remembering dates	−0.09	0.07	0.03
I	8 Events from years ago	−0.16	0.11	0.19*
II	3 Events from minutes ago	−0.19*	−0.10	− 0.20*
II	6 Planned activities	−0.16	−0.12	− 0.12
II	7 Events from days ago	−0.22*	−0.02	0.03
II	9 Keeping track	−0.23**	−0.23**	− 0.00

* = *p* < 0.05; ** = *p* < 0.01 one-tailed statistical significance. Significant results are bolded

## Discussion

There are three main findings in this study of subjective memory assessment (MMQ total, components and items) and its relationship with objective memory performance, age, education and gender in a sample of older adults. In discussing the findings, we deal primarily with empirical associations between subjective and objective memory in relation to demographics in older adults.

Firstly, as shown in Table 4, the results of a specific part of the MMQ, i.e., Component II (related to subjective ratings of impairments in attention and working/short-term memory), were significantly correlated with all three objective memory test scores i.e. objective episodic, semantic and working/short-term memory. This pattern was observed for three Component II items related to current or recent events (see Table 6), but not for any Component I item. It could be speculated that attention is a common factor to Component II and LM, as attention is an integral part of both Component II and theories of learning and memory [27]. The relationship between working memory (Component II) and LM indicates that subjective working memory (Component II) is more strongly related than subjective declarative memory (Component I) to objective memory performance. To the best of our knowledge, this pattern has not been shown previously.

Secondly, Component I (ratings related to long term/declarative memory of events, dates and names) was not significantly correlated with objective tests of episodic, semantic and working/short-term memory performance according to standard clinical memory tests (see Table 4). The regression analyses reported in Table 5, showed that subjective long-term/declarative memory (Component I) was positively associated with two objective tests (Voc and LNS); however, these findings are considered as spurious, because of unreasonable direction (the more impairment the better results). In fact, as Table 6 shows, none of the five items linked to subjective long-term/declarative memory (Component I) was significantly associated with objective memory performance. This means that a clear-cut pattern emerged from our correlations and regression analyses, indicating that subjective long-term/declarative memory (Component I), as well as items associated with Component I (e.g. memory problems, remembering dates), which at face value may seem to correlate with objective episodic and semantic memory tests, do not in fact do so, at least not in this sample of healthy older adults. A similar conclusion has been reported by other researchers [7]. Consequently, subjective memory assessment will give different outcomes from those attained with objective memory assessment and therefore both types of assessment may be needed. A second implication would be that to learn about a person’s episodic/semantic memory, you should ask about the person’s ratings of present activity and most recent events. In an earlier paper [23], for example, we showed that MMQ outcomes could be used to differentiate between demented and healthy older individuals.

Thirdly, education was a strong, significant predictor of all three objective memory tests (see Table 5). Several studies have reported that education is a significant moderating factor in objective cognitive function testing in older adults [31]. In this study, the power of education as a predictor was greater than the power of Component II. The effects of education have often been related to cognitive reserve [32], and research findings support the suggestion that education is one of the key factors preventing cognitive decline and dementia [33]. Regardless, because education is such a powerful moderator of several aspects of objective memory performance, it should be considered in clinical subjective and objective memory assessments.

In addition, age was a strong significant predictor of all three objective memory measures (see Table 5). This finding is well known in normal aging, as well as in clinical studies of patients with brain diseases. Interestingly, there is a paradoxical difference between age-related changes in objective memory tests, while there were no significant age-related changes in subjective memory measures in the present study (see results on correlations with demographics). A similar pattern of associations was observed for education, namely significant correlation between objective, but not subjective memory measures and education.

Furthermore, no association was found between gender and objective memory performance in the study sample, in contrast to the findings of Holmen et al. [22] that there were more subjective memory complaints in men than in women in the HUNT3 population. These divergent findings may be because Holmen et al. [22] based their study on the total HUNT3 population and only used the MMQ total score to subjectively assess memory. There were no significant associations between age and subjective memory performance, despite a strong, significant association between age and all objective memory test outcomes. This may be because, in subjective assessments of memory, one compares oneself with neighbors and friends of approximately the same age, where some have poor memory and some have good memory. In objective tests, however, memory performance is compared with that of the total population of various ages rather than with closely related individuals.

The MMQ total score together with age and education significantly predicted the LM, but not the VOC and LNS performance according to the multiple regression analyses, see results. The explanation for these complex associations may depend on the fact that three items related to Component II (3, 7 and 9) were significantly correlated to LM and one item (3) to LNS, see Table 6.

Older adults frequently worry about their memories. It is therefore essential that the subjective questionnaires used to assess memory also allow the documentation of the statistical characteristics associated with the results at hand, to ensure that health personnel can understand what the results of those questionnaires really mean, so they can convey the outcomes to the worried patients. This study showed that certain items in the MMQ are more informative of objective memory test outcomes than others, while other MMQ items may be more closely linked to aspects of ordinary life, such as emotions, personal stress, etc. [17]. This pattern of complex results indicates a differential relationship between specific items in component II (working/short-term memory) and performance in objective memory, which illustrates that objective memory tests may not be exclusively uni-dimensional. Previously it was shown that the MMQ is a multi-dimensional measure of subjective memory [20].

### Strengths and weaknesses

The primary advantage of this study is that the scores of different MMQ components, rather than a single subjective memory score, were compared to objective memory performance. A second advantage was that the domain-specific objective memory performance was assessed using well validated tests (WMS-III/WAIS-III). To our knowledge, this kind of study has not been done previously. Furthermore, except for age, the sample was comparable to the general HUNT3 population, indicating that the findings may also be extrapolated to the HUNT3 population. Finally, because the items of the MMQ are similar in content to many other tests of subjective memory, the findings in the present study may have general relevance.

One disadvantage of this study is that the sample was relatively small. Also, the subjective assessment of memory was made approximately 2 years before the objective assessment was made, which could have inflated the results of the MMQ. However, the analyses were repeated to control for the time interval. The pattern of results remained the same after this implying that the assumption of inflated results is less likely. The generalizability of the presented results may be limited and dependent on the similarity between the MMQ and other methods of subjective memory, as well as the similarity between the present cohort and cohorts in other studies.

## Conclusion

In cognitively normal older adults, working/short-term memory aspects of subjective memory assessments seem to be an indicator of objective episodic memory performance. Clinicians and researchers might consider targeting working/short-term memory aspects of subjective memory assessments when seeking an estimate of objective memory performance.

## Data Availability

The dataset used in this study is available on appropriate request to the Data Access Committee at the HUNT Research Centre (www.hunt.no). The data are not publicly available due to privacy protection based on Norwegian law and the consent of the participants.

## Abbreviations

HUNT3: Third wave of the North-Trøndelag Health survey
LM: Logical Memory subtest of the Wechsler Memory Scale-III
LNS: Letter-Number Sequencing subtest of the Wechsler Adult Intelligence Scale-III
MMQ: Meta-Memory Questionnaire
Q1: Questionnaire 1
Q2: Questionnaire 2
Voc: Vocabulary subtest of the Wechsler Adult Intelligence Scale-III
WAIS-III: Wechsler Adult Intelligence Scale-III
WMS-III: Wechsler Memory Scale-III
